# A mirror-image analysis of psychiatric hospitalisations among people with severe mental illness using Independent Supported Housing

**DOI:** 10.1186/s12888-022-04133-5

**Published:** 2022-07-22

**Authors:** Christine Adamus, Simeon Joel Zürcher, Dirk Richter

**Affiliations:** 1 Center for Psychiatric Rehabilitation, Universitäre Psychiatrische Dienste Bern (UPD), Bern, Switzerland; 2 University Hospital of Psychiatry and Psychotherapy, University of Bern, Bern, Switzerland; 3 Department of Health Professions, Bern University of Applied Sciences, Bern, Switzerland

**Keywords:** Independent Supported Housing, Mirror-image study design, Severe mental illness, Psychiatric hospitalisations

## Abstract

**Background:**

Evidence on the effectiveness of Independent Supported Housing (ISH) for non-homeless people with severe mental illness primarily comes from observational cohort studies, which have high risk of bias due to confounding by time-invariant sample characteristics. The present study proposes an alternative study design known from pharmacology to overcome this bias and strengthen evidence.

**Methods:**

We conducted a retrospective mirror-image analysis with medical records of 144 ISH service users to assess the effectiveness of ISH in reducing the number and duration of hospitalisations. Outcomes occurring in equal periods before and during ISH utilisation were compared for every ISH user. Differences between the periods were tested with incidence rate ratios (IRR).

**Results:**

Included service users were on average 38.2 years old, female (54%) and predominately had an affective (28.5%) or a schizophrenic or psychotic (22.9%) disorder with ISH utilisation days ranging from 36–960. Fewer admissions (IRR = 0.41, 95%-CI 0.27–0.64) and fewer person-days hospitalised (IRR = 0.38, 95%-CI 0.35–0.41) were observed during ISH utilisation compared to prior to their ISH utilisation. While the reduction in psychiatric admissions may be somewhat confounded by time-variant characteristics, the substantial reduction in hospitalised bed-bays represents at least partially an intervention effect.

**Conclusions:**

The mirror-image study design allowed for a cost-effective investigation of ISH effectiveness in reducing hospitalisation without confounding by time-invariant sample characteristics. We provide recommendations for the design’s application and suggest further research with larger samples.

## Background

Independent Supported Housing (ISH) interventions provide people with severe mental illness (SMI) with psychosocial support in their independent accommodation without the “treatment first” necessity to help them manage their mental illness and foster social inclusion and recovery. ISH interventions are considered evidence-based in the support of homeless service users and show promising results, especially regarding improvement of housing stability and reduction of psychiatric hospitalisations [1–4]. However, evidence on its effectiveness in supporting non-homeless persons is weak and the scarce results show mixed findings [2, 3, 5]. Moreover, the general feasibility to conduct randomised controlled studies (RCT) on housing settings for non-homeless people is limited [6]. Aside from ethical difficulties in randomly allocating housing conditions, strong preferences of service users for independent living [7] and high level of gatekeeping by staff [6] impede the conduction of randomised studies [8]. Currently, there is only one single RCT on the effectiveness of ISH for non-homeless service users, which was only feasible with limiting access to the intervention for study participants only [9].

Randomisation is considered as “gold-standard” for achieving balanced groups in intervention studies. Observational cohort studies, on the other hand, have high risk for self-selection bias due to non-random allocation methods [10]. Non-random allocation such as referral by clinicians or self-selection to study conditions is often influenced by participants’ characteristics, which in turn may be linked with the treatment or the outcome under investigation [11]. As a consequence, sample characteristics in non-randomised studies may systematically differ between study conditions and may confound the estimated treatment effects. There are several statistical attempts to control for multiple confounders in observational studies like regression adjustment [12] and propensity score methods [11]. Such statistical methods can mitigate the effect of confounding by adjusting for the observed sample characteristics. However, these methods cannot rule out confounding by non-measured characteristics as randomisation methods would [13]. Thus, existing observational studies on the effects of ISH in the support of non-homeless people may be supplemented by alternative study designs to foster evidence with less biased results [14].

One such design could be the mirror-image design, which is a self-controlled study design known from pharmacology. In mirror-image studies, outcomes occurring in a period before an index event (e.g., starting an intervention) are compared with the outcomes occurring in a period of equal length after the index event [15]. The mirror-image design has several advantages that make it a valuable complement to observational cohort studies. Firstly, no time-invariant characteristics of the included subjects confound the findings because each subject acts as its own control, thus called self-controlled [16]. Secondly, the retrospective investigation of routinely collected patient data allows for a cost-effective investigation of objective outcome variables (e.g., hospitalisations). Thirdly, with the use of routine data, no recruitment and participation of service users is required. Thus, no possible effects of a trial itself (allocation, assessments, etc.) may alter treatment delivery or bias the outcomes (e.g., no Hawthorne effect) [15, 17]. Additionally, it allows for inclusion of all eligible subjects in the analysis with no restriction to consenting participants, which improves representativeness [15]. This therefore allows for the conduction of effectiveness research on the intervention in a naturalistic setting and under real-world circumstances.

However, there are also design-inherent limitations. Time-varying aspects may confound results from mirror-image studies. For example, a change in outcomes may represent the natural course of the illness instead of an intervention effect, and therefore, a regression toward the mean may bias the results [15, 18]. Because each subject’s outcome is compared before and after the start of an intervention, and because the start of a new treatment is never a random event but rather is initiated due to a specific, outcome-related cause (e.g., follow-up treatment after hospital discharge, illness exacerbation), it is important to account for time-varying aspects in the implementation and interpretation of mirror-image studies [15].

The present study aimed to apply the mirror-image design to study the effects of ISH in reducing the number and duration of hospitalisations in a sample of non-homeless ISH users with SMI while considering possible bias.

## Methods

### Study design

We conducted a retrospective mirror-image study with anonymised medical record data of ISH service users. The individual start date of ISH was defined as index. Number of events defined below (see outcomes variables) were compared between post-index (during intervention) and pre-index (before intervention) periods of equal length.

### Intervention

ISH is a community-based outreach housing rehabilitation service provided since 2012 by the Centre for Psychiatric Rehabilitation of the University Hospital of Psychiatry in Bern, Switzerland [19]. It follows the principles of the “Housing First” approach [20] as it is independent of service users’ therapy and care and is not transitional, but permanent and without time limitation. ISH addresses adult people with SMI and provides its users with psychosocial support in their independently rented accommodation. The main goals of the ISH intervention is the social inclusion of its users, including fostering their autonomy and personal recovery. According to the Simple Taxonomy for Supported Accommodation (STAX-SA; [21]) the intervention classifies as a Type 4 service as service users live in individual accommodations with no staff on-site and the intervention provides low to moderate support with no time-limitation.

Support services are provided up to 8 h per week by non-medical staff with nursing or social work training. An offsite residential coach supports the service users according to their needs in all aspects related to finding and keeping ones’ accommodations. This may include contacts with the landlord, social environment, administration, and cooperation with mental health services. Service users also have the option to consult an ISH psychiatrist. The psychiatric, psychotherapeutic and medical treatment of service users takes place outside the ISH service by appropriate specialists.

Model fidelity of the ISH intervention was assessed in 2019 as part of another study [9] using the MSSW model fidelity scale (*Modelltreue-Skala Selbstbestimmtes Wohnen* [Independent Supported Housing Fidelity Scale]; [22]). Fidelity was high with 141 out of a possible 155 total sum score (*m* = 4.5) and subscale scores of *m* = 4.67 (housing conditions), *m* = 4.5 (staff/team), *m* = 4.4 (support conditions), and *m* = 4.83 (inclusion orientation) out of a possible mean score of five.

### Sample

From the medical records, we extracted every ISH utilisation period with start date between July 2^nd^ 2016 and February 28^th^ 2019. The start date was chosen because the patient medical records were retrospectively introduced into the medical records system on July 1^st^ 2016 and therefore lack some information about ISH utilisation before this implementation. The end date was chosen because an augmented Home Treatment/Crisis Resolution Treatment program was implemented in the ISH programme in March 2019. We included all ISH utilisation periods within this time window, if it was the service user’s first utilisation. If a service user had multiple ISH utilisations within this time window, we included the first utilisation period and excluded the latter. In addition, ISH utilisation periods of less than 30 days (all of them had a start date after January 2019) were excluded to increase the probability that included service users actually received support within the observation period. The included ISH utilisation periods are either limited by withdrawal from the program (case finalisation for any reasons) or by censoring on February 28^th^ 2019 in case of ongoing ISH use.

The cantonal ethics committee of Berne, Switzerland reviewed the study and confirmed that approval of an institutional review board was not required (Req-2021–00042, January 2021).

### Measures and source of information

#### Outcome variables

Outcome data was retrieved from patient medical records of the psychiatric hospital. Outcome variables were extracted for each pre- and post-index mirror-image period defined below (see statistical methods) and include the number of inpatient psychiatric admissions and the length of inpatient psychiatric hospital stays, defined as the number of person-days hospitalised (including censored stays, e.g., with admission before a mirror-image period’s start).

#### Sample characteristics

The medical records were used to retrieve sample characteristics and consist of service users’ demographic information: age (in years), sex (female, male), nationality (Swiss vs. non-Swiss), and civil status (single vs. married, divorced, widowed). Clinical information of the main psychiatric diagnosis category according to the ICD-10 classification of mental and behavioural disorders [23] was also obtained.

### Statistical methods

Sample characteristics and hospitalisation patterns are reported descriptively.

The primary analysis was a mirror-image analysis of the outcome measures defined above. Post-index outcomes were compared with pre-index outcomes in each mirror-image period. The maximum period length for each service user was defined as the individual ISH utilisation period as described above (see sample). In addition, we defined mirror-image periods of 90, 180, 270, and 365 days to assess the possible influence of the different utilisation period lengths. Service users could be included in several mirror-image periods if their utilisation period covered the entire period. The change of psychiatric hospitalisations from pre- to post-index was analysed by computing incidence rate ratios (IRR) with 95% confidence intervals (95%-CI). In line with other self-controlled studies [24, 25], a simple analysis was conducted that did not account for the fact that users were observed under two conditions (before and after ISH initiation). This approach was adopted to circumvent the issue of zero events i.e. in psychiatric admissions. This is a conservative approach that exaggerates the magnitude of standard errors [15].

We further conducted sensitivity analyses to address the potential of a regression towards the mean [14, 15, 18], which could have led to an overestimation of ISH effects. Since this bias is assumed to be more strongly affected by pre-index outcomes occurring close to the index rather than by long-term outcomes [26], we reanalysed every mirror-image analysis after excluding all ISH users who had a psychiatric admission within 90 days before index.

All statistical analyses were performed with R version 4.0.3 [27] and the *fmsb* package for computing IRR [28]. Statistical significance was set at *p* < 0.05 for all analyses.

## Results

### Descriptive results

**Fig. 1:**
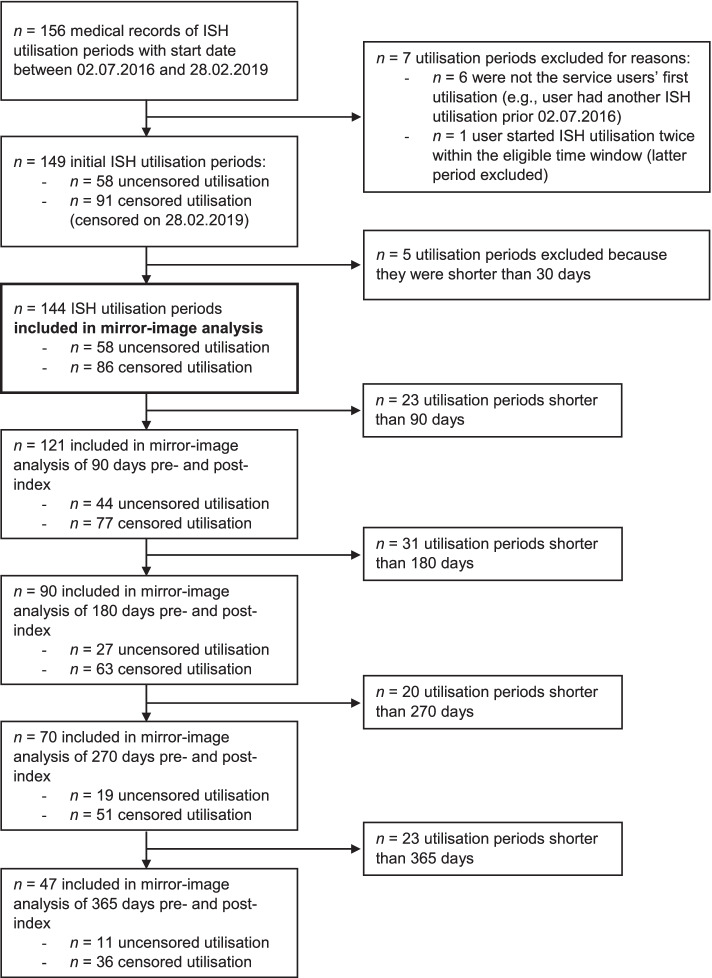
Flow chart of the inclusion and exclusion process of the ISH utilisation periods. Inclusion of ISH utilisation periods and defined mirror-image periods of 90, 180, 270 and 365 days, each with information on censoring status. Utilisation periods were limited either by withdrawal from program (uncensored utilisation) or by censoring on February 28^th^ 2019 in case of ongoing ISH use. ISH: Independent Supported Housing

One hundred fifty-six ISH utilisation periods of 155 service users were extracted. One service user utilised ISH twice within the eligible time window, and the latter utilisation period was excluded. Six utilisation periods were excluded because the service users had another ISH utilisation before July 2016. Five utilisation periods were excluded because the observation period was less than 30 days (all of them started in January 2019). The inclusion and exclusion process of ISH users’ utilisation periods, the procedure of the inclusion in the defined mirror-image periods and information on censoring status in each mirror-image period are shown in the flow chart in Fig. 1.

The sample included utilisation periods of 144 initial ISH users aged between 17 and 64 years (*m* = 38.2, *SD* = 13.3) at the start date of ISH (Table 1). The majority of the service users were female (54%), single (82%), had an affective (28.5%) or a schizophrenic or psychotic disorder (22.9%) and were of Swiss nationality (96%).

**Table 1:** Demographic and clinical characteristics at the ISH start date (mirror index; *n* = 144)

**Age, mean (*SD*)**	Years	38.2 (13.3)
**Sex, *N* (%)**	Female	78 (54.2)
**ICD-10 Diagnosis, *N* (%)**	F1: Substance abuse disorder	15 (10.4)
	F2: Schizophrenic or psychotic disorder	33 (22.9)
	F3: Affective disorder	41 (28.5)
	F4: Neurotic, stress and somatoform disorder	15 (10.4)
	F6: Personality disorder	20 (13.9)
	Other psychiatric disorder	20 (13.9)
**Nationality, *N* (%)**	Swiss	138 (95.8)
**Civil status, *N* (%)**	Single	118 (81.9)
	Divorced / widowed	18 (12.5)
	Married	8 (5.6)

*N* Number of service users, *SD* Standard deviation, *ISH* Independent Supported Housing. “Other psychiatric disorders” contain F0: organic diagnoses (*n* = 5), F8: developmental disabilities (*n* = 6), F9: disorders with onset in childhood and adolescence (*n* = 8) and unknown diagnosis (*n* = 1)

The length of the included ISH utilisation periods varied between 36 and 960 days with a mean duration of 310.3 days (*SD* = 228.8; median = 266.5 days). In sum, the utilisation periods covered 44,690 person-days, or 122.4 person-years. The utilisation periods of 86 (59.7%) service users were coded up to February 28^th^ 2019 (censored utilisation period), and 58 (40.3%) users stopped using ISH before this date (uncensored utilisation period). The uncensored utilisation periods ranged from 36 to 788 days with a mean utilisation duration of 216.6 days (*SD* = 161.8; median = 159.5 days). Four of the 58 uncensored ISH utilisations ended during a psychiatric hospitalisation (utilisation periods: 36, 51, 62, 171 days).

### Mirror-image analyses

**Fig. 2:**
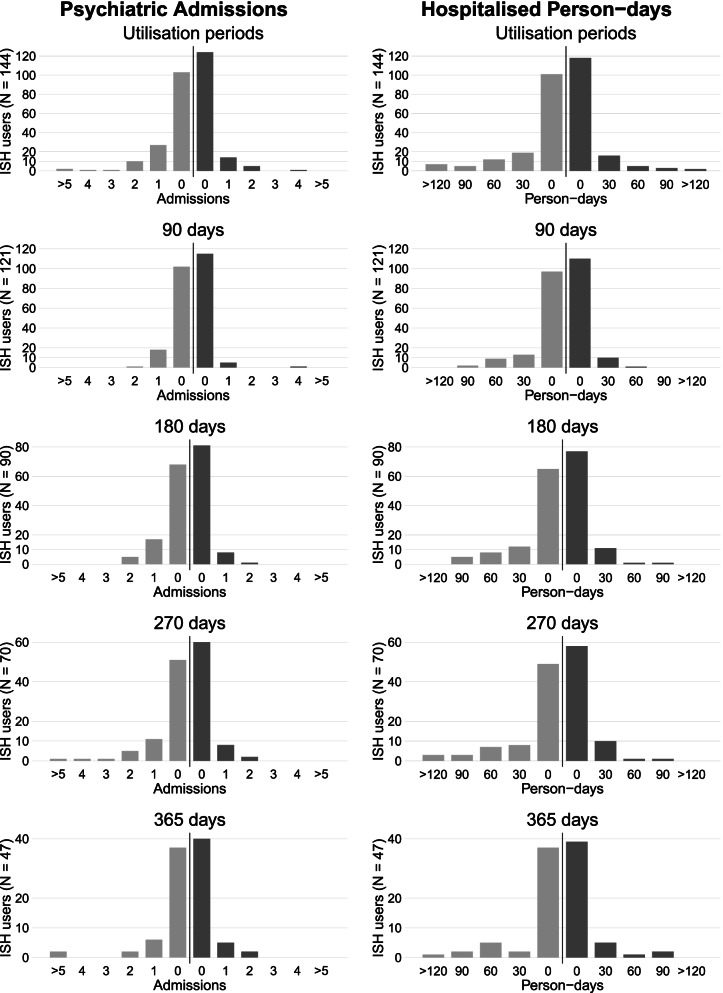
Frequencies of psychiatric admissions and hospitalised person-days in all mirror-image periods. Number of psychiatric admissions and hospitalised person-days before (bright grey) and after (dark grey) ISH initiation (index)

**Fig. 3:**
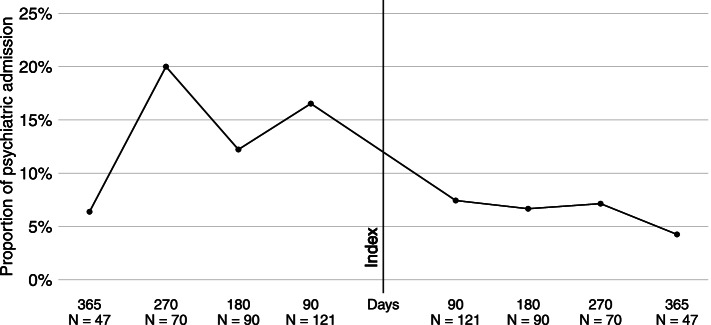
Proportion of psychiatric admissions within each 90-days interval before and after ISH initiation (index)**.** Intervals of 90 days show proportions of admissions within each quarter year (e.g., the 180 days with ninety service users including proportional number of admissions within 91 and 180 days)

Figure 2 presents the distribution of the psychiatric admissions and hospitalised person-days showing more hospitalisations per person and longer hospitalisation stays before ISH than after its implementation. Figure 3 shows the proportion of hospital admissions within separate 90-day intervals before and after ISH implementation. The proportion of admissions ranged from 6 to 20% and from 4 to 7.5% before and after ISH implementation, respectively. There were consistently fewer post-index hospitalisations than pre-index. The proportional number of admissions was highest within the 90-day interval from 181 to 270 days before ISH. A second increase in hospitalisations occurred close to the index. The risk of bias due to this second increase was considered in the sensitivity analyses, for which we excluded all the 24 ISH users who had at least one psychiatric admission within 90 days before ISH.

The IRR of the mirror-image analyses are shown in Table 2. Results indicate significantly reduced incidences of psychiatric hospitalisations in both outcome measures and in all mirror-image periods. After ISH initiation, the incident rate of admission ranged from 0.36 (95%-CI: 0.19–0.70) to 0.45 (95%-CI: 0.20–0.99) times the pre-index admission rate across all mirror-image periods. Similarly, the IRR showed a reduction of person-days hospitalised after the index in the range of 0.19 (95%-CI: 0.16–0.23) and 0.43 (95%-CI: 0.37–0.50) across all mirror-image periods. The sensitivity analyses resulted in substantially reduced effect sizes and larger confidence intervals and yielded non-significant results regarding the number of inpatient psychiatric admissions. The reduction in hospitalised person-days was still significant with an IRR ranging from 0.13 (95%-CI: 0.09–0.20) to 0.59 (95%-CI: 0.49–0.71) in all but the 365-day period.

**Table 2:** Mirror-image analysis of inpatient psychiatric hospitalisations

	**Outcome**	**Mirror-image period**	**Sample size (*n*)**	**pre-index (*n*)**	**post-index (*n*)**	**IRR**	**95% CI**	
							**upper**	**lower**
**Primary Analysis**	No. of psychiatric admissions	utilisation period	144	68	28	**0.41**	0.27	0.64
		90 days	121	20	9	**0.45**	0.20	0.99
		180 days	90	27	10	**0.37**	0.18	0.77
		270 days	70	33	12	**0.36**	0.19	0.70
		365 days	47	21	9	**0.43**	0.20	0.94
	No. of psychiatric person-days (including censored stays)	utilisation period	144	2113	802	**0.38**	0.35	0.41
		90 days	121	787	149	**0.19**	0.16	0.23
		180 days	90	908	229	**0.25**	0.22	0.29
		270 days	70	939	233	**0.25**	0.21	0.29
		365 days	47	512	219	**0.43**	0.37	0.50
**Sensitivity Analysis**	No. of psychiatric admissions	utilisation period	120	26	21	0.81	0.45	1.44
		90 days	102	0	3	-	-	-
		180 days	74	7	7	1.00	0.35	2.85
		270 days	58	10	10	1.00	0.42	2.40
		365 days	42	6	9	1.50	0.53	4.21
	No. of psychiatric person-days (including censored stays)	utilisation period	120	1089	622	**0.57**	0.52	0.63
		90 days	102	225	30	**0.13**	0.09	0.20
		180 days	74	299	177	**0.59**	0.49	0.71
		270 days	58	369	176	**0.48**	0.40	0.57
		365 days	42	230	211	0.92	0.76	1.11

Primary and sensitivity analyses are conducted with whole utilisation periods and mirror-image periods of 90, 180, 270 and 365 days post- vs. pre- ISH initiation (index). The absolute number of outcome variables occurring in each pre- and post-index period are compared by IRR and are tested for significance by 95% CI. In sensitivity analyses, people were excluded if they had an admission within 90 days before index. Censored stays resulted from hospitalisations with admission before the observation period started

*N* Number of service users included in the analysis, *IRR* incidence rate ratio, *CI* confidence interval, *ISH* Independent Supported Housing

## Discussion

We conducted a mirror-image study on the effectiveness of ISH in reducing psychiatric hospitalisations of service users with SMI by comparing the number and duration of hospitalisations within equal periods during (post-index) vs. before (pre-index) their ISH utilisation. The results showed the included 144 ISH users were significantly less likely to utilise psychiatric hospitalisation treatment compared to prior to their ISH utilisation. This decline in hospitalisations during ISH utilisation occurred in both outcomes and in all analysis periods. However, after excluding those with a pre-index hospitalisation occurring shortly before the ISH start in our sensitivity analyses, the number of psychiatric admissions did not change significantly. Therefore, it is possible that functional improvements independent from the ISH intervention affected the decrease in psychiatric admissions, resulting in a regression towards the mean phenomenon instead of an intervention effect [15, 18, 26]. Nonetheless, the overall reduction in hospitalised bed-days was substantial and significant in every mirror-image period in the primary and in most periods of the sensitivity analyses, and individual hospitalisation durations were shorter during ISH (Fig. 2).

In line with our results, we found one study that compared periods of two years prior and during the initiation of a supported housing programme for non-homeless people with SMI in Australia [29]. This comparison showed both a reduction in the number of admissions and in the mean length of hospitalisation stays during the supported housing use. However, shortly before the programme started there was a strong increase in hospitalisations, and long-term differences were much smaller. Therefore, it is possible that their results at least partially reflect a regression towards the mean instead of an intervention effect. Nevertheless, the length of hospitalisation stays substantially declined during the intervention and this decline was even stronger with regard to long-term outcomes.

Our findings are also in line with the evidence on hospitalisation reduction with different forms of permanent/independent supported housing for people with SMI, which is mixed regarding both homeless and non-homeless populations. One systematic review showed a significant reduction of hospitalisation use in homeless persons with permanent supportive housing in most studies [1]. Similarly, a meta-analysis showed reduced hospitalisations in homeless persons with all housing models in contrast to non-model housing (housing simply described as “treatment as usual”) but no differences between the housing models (e.g. permanent supported housing, residential care and treatment, residential continuum) [30]. Another systematic review also showed mixed findings with some positive findings and other studies reporting no change in hospitalisation use regarding both homeless and non-homeless populations [2]. However, the herein reported findings emerge from a wide variety of different housing support models not limited to permanent/independent supported housing models. Similarly, a systematic review on ISH outcomes reported equal or reduced hospitalisation use in homeless populations. Regarding non-homeless service users living in independent accommodations, one study showed equal hospitalisation use and one study reported increased hospitalisations or healthcare utilisation [3]. However, the latter finding was found in forensic patients not receiving outreaching support [31].

The mirror-image design implemented here showed several advantages in comparison to (non-) randomised cohort studies that supports this design as a valuable complement to the existing evidence on ISH. It allowed for an inclusion of every service user, and service users did withdraw only from the intervention and no study dropouts occur in the sample. Consenting and non-discontinuing participants with SMI, in turn, are assumed to show better functional status, therefore people with low treatment adherence, low functioning or severe substance abuse problems may be underrepresented in cohort studies [15]. This analysis included a more representative sample of the intervention’s target population without a restriction to consenting participants. Additionally, cohort studies may alter treatment delivery (e.g., more frequent consultations, reminders) [15, 17] or participation (pre-treatment motivation, in-treatment compliance) [8, 10]. This mirror-image study investigated the intervention effects under real-world circumstances in a cost-effective manner and on an objective outcome. Because the mirror-image design investigates outcome events occurring within defined periods (vs. assessment points), outcome data in mirror-image analyses is limited to routinely and completely collected data. Therefore and as a disadvantage of the design, investigating the effectiveness of ISH in fostering its service users’ quality of life, social inclusion, physical or mental wellbeing or housing situations is usually not possible with mirror-image studies. Nonetheless, there was no confounding due to time-invariant characteristics of included subjects, as each service user in the analysis served as her or his own control. Therefore, the mirror-image design overcomes the main drawback of existing evidence on ISH effectiveness resulting from self-selected allocation to either condition in non-randomised cohort studies.

### Limitations

The present study has some limitations to take into consideration when interpreting the results. Firstly, the sample size was small, especially in the longer mirror-image periods. Secondly, there was possibly some missing outcome data since the available data source did not contain information about potential hospitalisations in other psychiatric hospitals. However, because the ISH intervention arrange hospitalisations for its service users predominantly in the investigated psychiatric hospital, this limitation would have affected the results in a conservative manner. The development of large nationwide datasets would improve studies on patient medical records data. Thirdly, we computed IRR with a simple approach that did not incorporate repeated outcome measures. Therefore, this statistical method likely led to overestimate confidence intervals due to an exaggeration of standard errors [15]. We further did not conduct regression analyses investigating factors related to the number and/or duration of hospitalisations. There is still great uncertainty on characteristics predicting hospitalisation use beyond clinical severity and crises [32–34]. Gaining insight into such predicting factors would help tailor ISH for even better prevention of psychiatric hospital admissions. Finally, ending the observation period after intervention withdrawal brings some risk of selection bias, which would be in favour of the investigated intervention if the reason for stopping the intervention remains unassessed [15]. In our analyses, four out of the 58 uncensored cases had a hospitalisation during withdrawal from intervention. All of these cases had rather short utilisation periods and therefore, risk of selection bias was higher in shorter periods.

## Conclusion

The mirror-image design in this study was cost-effective and practical for investigating the effectiveness of ISH in a population of non-homeless service users. ISH service users showed substantially less bed-days in a psychiatric hospital than in an equal period preceding their ISH use. They also showed fewer psychiatric admissions during ISH than before they started with ISH. However, because ISH could be implemented as a follow-up treatment after (pre-index) psychiatric discharge to ensure daily support after inpatient treatment, the reduced admissions could also reflect service users’ natural course of disease (regression towards the mean) instead of an intervention effect.

Further applications of the mirror-image study design on ISH effectiveness in supporting non-homeless people are suggested to give special caution to two sources of possible design-inherent biases, which both would work in favour of the treatment under investigation. Mirror-image design-inherent biases either are related to the index definition as the intervention start if pre-index outcomes were not adequately handled in the analyses (regression towards the mean) [14, 15, 18, 26], or are related to the definition of the mirror-image periods’ length if outcomes occurring immediately after the post-index observation period were ignored (selection bias) [15]. Two additional suggestions could further improve mirror-image studies on ISH. Firstly, bi-directional mirror-image analyses may reduce the effect of the discussed biases [15, 26]. Bi-directional mirror-image analyses contain subjects using ISH in the post-index period (index: intervention start) and other subjects using ISH in the pre-index period (index: intervention withdrawal). This would help for a better control of time-effects like the natural course of disease [26]. Secondly, to prevent selection bias, future mirror-image studies are suggested to define periods of a prespecified length and on an intent-to-treat basis including all subjects even after discontinuation [26].

Considering these aspects, the mirror-image design brings many advantages that make the design a valuable complement, improving existing evidence on the effectiveness of ISH in non-homeless service users.

## Data Availability

The datasets generated and analysed during the current study are not publicly available because they were completely driven from patient medical records.

## Abbreviations

ICD-10: International Classification of Diseases, 10th edition
IRR: Incidence Rate Ratio
ISH: Independent Supported Housing
SMI: Severe mental illness
